# The Illinois State Board of Health

**Published:** 1885-08

**Authors:** 


### STATE BOARDS OF HEALTH.

THE ILLINOIS STATE BOARD OF HEALTH.

The Illinois State Board of Health held its regular quarterly meeting in Chicago on the 2nd and 3rd of July.

In addition to the transaction of its usual routine business, the board proceeded to the consideration of the cases of those against whom charges of unprofessional conduct had been preferred. The first was that of John Bate, who was charged with having advertised his calling under such fictitious names as A. G. Olin and J. G. Carlton, and with having been convicted in the United States court of having sent obscene literature through the mails, for which he was sentenced to the penitentiary. Proper notice was served upon him prior to the meeting of the board, but he failed to appear, either in person or by counsel, to defend himself against these charges, which were considered in due form by the board. The evidence of the correctness of the charges preferred seemed conclusive to the board, and the license to practice medicine, which had been issued to him in 1877, was revoked and annulled, on the ground of his having been "guilty of unprofessional and dishonorable conduct."

The next case considered was that of one J. Cresap McCoy, now located in St. Louis, Missouri, who had once been located in Belleville, Illinois. He was charged by the board with having circulated his advertisements, in which claims and promises were made by him which were considered false and fraudulent. He was duly notified to appear before the board to defend himself against the charges made against him. He failed to appear, as required, either in person or by counsel, and after having investigated his case the board considered the charges fully proven and revoked and annulled the license, "for unprofessional and dishonorable conduct," which had been issued to him in February, 1885.

Other cases were considered, among them that of "one Henry H. Williams," of St. Louis; also of "a Dr. Westmoreland," of St. Louis, and of a "J. G. Gilfilan," at Lenzburg, Illinois, and of "H. D. Flower, M. D., of Fulton City," Illinois, and of "Dr. W. B. Watson, of Streator, Illinois." Their cases have not yet been disposed of by the board.

The report of the secretary of the board makes a very favorable showing of the public health of the State of Illinois.

A few scattered cases of small-pox have occurred during the preceding three months, in different parts of the State, but the disease had been prevented from spreading.

The board has not relaxed its efforts at securing good sanitation for the entire state, which were instituted when the danger of the appearance of cholera in this country seemed greater than it does at present. Even in the absence of this dreaded scourge, the good accomplished—immediate and prospective—by such work is of incalculable advantage to the individual and to the state, and the board deserves recognition of its efforts, and commendation for them.

The secretary's report notices the fact that the city of Chicago has placed an additional appropriation of one hundred thousand dollars at the disposal of the Commissioner of Health of the city, for the improvement of the sanitary condition of the city, which sum is being judiciously expended, and the house-to-house inspection thoroughly conducted. The legislature of the state has made an appropriation of forty thousand dollars as a contingent fund, available in case of an outbreak of any epidemic disease. In addition, it has increased the amount of the appropriation for the current expenses of the State Board of Health.

The subject of the existence in the state of cases of glanders was considered by the board, and a conference was had with the State Veterinarian, who strongly urged upon physicians and the public generally, the exercise of great care regarding this fatal disease. Horses affected with it should be killed without consideration to their pecuniary value. On no account should any chances be taken of the disease being contracted by human beings, of which there is much danger, as may also be said of its kindred disease, known as farcy.

The secretary, in commenting on the report of the State Veterinarian, says: "The practical deduction from the foregoing is, that in localities where glanders among horses exists, physicians, in making up the diagnosis of an obscure case among their patients, would do well to bear in mind the resemblance of glanders, in the early stages, to rheumatism, typhoid fever, and pyæmia; and, in the chronic stage, to syphilis and tuberculosis. Under such circumstances, the occupation and history of the sufferer should be taken into consideration; and, remembering the highly contagious nature and practical incurability of glanders, proper precautions should be observed until it is demonstrated that it is not that disease."

